# Editorial: Efficacy and mechanism of herbal medicines and their functional compounds in preventing and treating cardiovascular diseases and cardiovascular disease risk factors-Volume II

**DOI:** 10.3389/fphar.2025.1742260

**Published:** 2025-12-09

**Authors:** Tiantian Meng, Yuqing Zhang, Jie Wang, Chen Huei Leo, Zhongfeng Li, Jian Zhang, Kuo Gao, Qingyong He

**Affiliations:** 1 Department of Rehabilitation, Dongfang Hospital, Beijing University of Chinese Medicine, Beijing, China; 2 Department of Cardiology, Guang’anmen Hospital, China Academy of Chinese Medical Sciences, Beijing, China; 3 Department of Oral and Maxillofacial Surgery/Pharmacology, School of Dental Medicine, University of Pennsylvania, Philadelphia, PA, United States; 4 Department of Health Research Methods, Evidence, and Impact, McMaster University, Hamilton, ON, Canada; 5 Department of Science, Mathematics and Technology, Singapore University of Technology and Design, Singapore, Singapore; 6 Department of Chemistry, Capital Normal University, Beijing, China; 7 Shanghai Frontiers Science Center of Optogenetic Techniques for Cell Metabolism, Shanghai Key Laboratory of New Drug Design, School of Pharmacy, East China University of Science and Technology, Shanghai, China; 8 School of Traditional Chinese Medicine, Beijing University of Chinese Medicine, Beijing, China

**Keywords:** cardiovascular diseases, cardiovascular disease risk factors, herbal medicine, therapeutic mechanisms, clinical efficacy

## Introduction

According to the latest statistics, cardiovascular diseases (CVDs) continue to be the primary cause of morbidity and death globally (Li et al., 2025; Mensah et al., 2023). Despite significant advancements in conventional pharmacotherapy and interventional strategies, the global burden of CVDs and their associated risk factors—such as diabetes, hypertension, and dyslipidemia—continues to rise (Global Burden of Cardiovascular Diseases Collaborators, 2025). Between 2025 and 2050, projections suggest a 90.0% increase in CVD prevalence, a 73.4% rise in the crude mortality rate, with deaths expected to reach 35.6 million, highlighting the critical requirement for alternative or complementary therapeutic strategies (Chong et al., 2025). Herbal medicines, with a history of thousands of years in traditional medical systems, have emerged as promising candidates for their multi-component, multi-target characteristics, and relatively favorable safety profiles compared to certain synthetic drugs (Yan et al., 2023).

In recent decades, increasing evidence from preclinical and clinical studies has illuminated the efficacy of herbal medicines and their bioactive compounds in modulating key pathological processes underlying CVDs, such as inflammation, oxidative stress, endothelial dysfunction, and myocardial remodeling (Cao et al., 2024; Huang et al., 2025; Yan et al., 2023; Zhang et al., 2025). Nonetheless, systematic integration of these fragmented findings and a thorough investigation of their molecular mechanisms are crucial for translating traditional knowledge into evidence-based clinical practice. To address this gap, we organized the Research Topic.

A total of 76 manuscripts were received, and after a rigorous peer-review process, this Research Topic successfully compiled 14 high-quality articles, encompassing original research studies and comprehensive reviews.

## Dyslipidemia

Dyslipidemia, commonly referred to as hyperlipidemia, is primarily defined by elevated levels of total cholesterol (TC, ≥200 mg/dL), triglycerides (TG, ≥150 mg/dL), and low-density lipoprotein cholesterol (LDL-C, ≥160 mg/dL) in the bloodstream, or by reduced levels of high-density lipoprotein cholesterol (HDL-C, <40 mg/dL in male or <50 mg/dL in female). This condition is recognized as a significant risk factor for CVDs (Zeljkovic et al., 2024).

Shi et al. performed a meta-analysis of 33 randomized controlled trials (RCTs) to evaluate the clinical efficacy of red yeast rice-containing Chinese polyherbal preparations in the treatment of dyslipidemia. The pooled results indicated that the combined preparations significantly reduced TC, TG, and LDL-C, while increasing HDL-C compared to statin therapy alone. Tian et al. reviewed the clinical efficacy and mechanisms of action of the classic traditional Chinese medicine (TCM) prescription Erchen decoction in treating hyperlipidemia. Previous clinical studies have shown that Erchen decoction and its modified formulations can reduce the levels of TG, TC, and LDL-C. Mechanistically, Erchen decoction primarily enhances lipid metabolism, regulates oxidative stress, and suppresses inflammation. Its active components mainly include β-sitosterol, *Poria cocos* polysaccharides, glycyrrhetinic acid, gingerol, oleanolic acid, and ursolic acid. Tang et al. systematically investigated the active ingredients and mechanisms of the BuShao Tiaozhi capsule for hyperlipidemia treatment. The findings indicated that BuShao Tiaozhi capsule effectively alleviates lipid metabolism disorders by blocking the Phosphatidylinositol 3-Kinase/Protein Kinase B (PI3K/Akt) pathway.

## Hypertensive heart disease

Hypertensive heart disease (HHD) is a secondary cardiac condition characterized by pathological alterations in cardiac structure and function, resulting from prolonged exposure to hypertension. It represents one of the most prevalent forms of target organ damage of hypertension and may eventually progress to heart failure (Díez and Butler, 2023).

An SR conducted by Hui et al. encompassed 21 RCTs, aiming to evaluate the clinical efficacy of specific TCM interventions designed to replenish qi and activate blood circulation as adjunctive treatments for hypertensive heart disease. The findings revealed that the combination of TCM and western medicine outperforms western medicine alone in enhancing cardiac function and alleviating adverse left ventricular remodeling. Notably, Danshen, Chuanxiong, Gegen, Huangqi, and Puhuang are the five most frequently utilized Chinese herbal medicines.

## Coronary heart disease

Coronary heart disease (CHD), commonly referred to as ischemic heart disease, includes acute coronary syndrome (such as unstable angina pectoris and myocardial infarction) and chronic coronary syndrome. It represents the leading cause of age-standardized mortality globally (Virani et al., 2023; Rao et al., 2025).

Six systematic reviews (SRs) have examined the effectiveness of herbal medicines in treating CHD. Wang et al.’s SR found that the modified Danggui Sini decoction, as an adjunct treatment, can shorten the duration of angina pectoris attacks, lower N-terminal pro-B-type natriuretic peptide levels, and improve the Seattle Angina Questionnaire scores in patients with CHD. Dai et al. performed a network meta-analysis comparing the clinical efficacy of ten different Danshen class injections for treating CHD. Their findings revealed that the combination of Danshen class injections with western medicine outperformed the use of western medicine alone. Specifically, Danshenduofensuanyan injection and Danshenchuanxiongqin injection demonstrated superior anti-inflammatory effects, while Danhong injection exhibited greater antioxidative properties. An SR by Chen et al. reported the efficacy of Salvianolate for injection, a Danshen-derived metabolite, as an adjunctive therapy for acute myocardial infarction. The pooled results from 30 RCTs involving 3,931 cases indicated that the combined use of Salvianolate for injection alongside western medicine could significantly decrease major adverse cardiac events incidence. An SR by Zhou et al., including 113 RCTs and 10,779 cases, aimed to evaluate the clinical efficacy of four TCM injections for tonifying qi in treating acute myocardial infarction. Findings indicated that combining these injections with conventional treatment decreased mortality and malignant arrhythmia risk in such patients, while also demonstrating enhanced safety. In a separate SR, Yu et al. compared the efficacy of the Guanxinshutong capsule alongside western medicine against western medicine alone for the treatment of stable angina pectoris. The results indicated that combining the Guanxinshutong capsule with western medicine can enhance electrocardiogram readings, left ventricular ejection fraction, and TC levels in individuals suffering from stable angina pectoris. Additionally, Mao et al. conducted a SR that included 28 preclinical studies to evaluate the efficacy and mechanism of Hydroxysafflor yellow A for ischemic heart disease. The results showed that Hydroxysafflor yellow A can reduce the area of myocardial infarction, lower the level of myocardial enzymes, and improve cardiac function. Its mechanisms may involve anti-inflammatory effects, anti-apoptosis, autophagy regulation, antioxidation, promotion of angiogenesis, and improvement of microcirculation.

## Myocardial ischemia-reperfusion injury

Myocardial ischemia-reperfusion injury (MIRI) is defined as the cellular damage that occurs when blood flow is restored to the ischemic heart, rather than facilitating functional recovery (Heusch, 2024).

Yang et al. conducted a meta-analysis of 32 animal experiments, demonstrating that salvianolic acid B can improve MIRI through mechanisms such as anti-inflammatory effects, antioxidation, reduction of apoptosis, regulation of vascular function, and promotion of angiogenesis. However, the efficacy of traditional therapies is often limited by suboptimal drug bioavailability, primarily attributable to poor target specificity. The review by Shi et al. summarized the integration of plant-derived secondary metabolites with nanotechnology as an innovative therapeutic strategy for MIRI. It highlights that nanocarriers, including liposomes, polylactic acid/glycolic acid nanoparticles, and mesoporous silica nanoparticles, enhance the stability, targeting precision, and bioavailability of plant-derived secondary metabolites, such as notoginsenosides, curcumin, and puerarin. This enhancement amplifies their antioxidant, anti-inflammatory, and anti-apoptotic effects, thereby mitigating myocardial damage associated with MIRI.

## Heart failure

Heart failure is characterized by impaired cardiac pumping function and represents the terminal stage of CVDs, serving as one of the leading causes of death (Bozkurt et al., 2025; Heidenreich et al., 2022).

In both cellular models, specifically angiotensin II-induced hypertrophic H9c2 cells, and animal models, such as a rat model of transverse aortic constriction-induced heart failure, Xu et al. demonstrated that the combined administration of Astragalus mongholicus and Salvia miltiorrhiza can enhance the prognosis of heart failure by inhibiting ferroptosis. The mechanisms underlying this effect may be linked to elevated levels of dihydroorotate dehydrogenase, ferroptosis suppressor protein 1, and glutathione peroxidase 4.

## Oxylipins

Oxylipins, which are bioactive lipid mediators oxidized from polyunsaturated fatty acids, are key regulators of inflammation, platelet aggregation, and vascular endothelial dysfunction—core processes driving cardiovascular pathologies such as atherosclerosis and hypertension (Ağagündüz et al., 2024).

A review by Li et al. systematically investigated oxylipins as critical biomarkers and mediators in CVDs, detailing their dysregulation in conditions such as hypertension, myocardial infarction, and heart failure. It further elucidated how TCMs modulate these oxylipin profiles; for example, they upregulate cardioprotective epoxyeicosatrienoic acids while downregulating pro-inflammatory hydroxyeicosatetraenoic acids, thus providing cardioprotection.

## Conclusion

This Research Topic successfully compiles 14 high-quality studies that advance our understanding of herbal medicines in addressing CVDs and their risk factors. Covering key conditions—from dyslipidemia and hypertensive heart disease to heart failure and MIRI—this Research Topic integrates SRs, meta-analyses, and mechanistic investigations to validate efficacy (e.g., improved lipid profiles, enhanced cardiac function) and unravel critical mechanisms, such as PI3K/Akt pathway modulation, ferroptosis suppression, and the use of nanotechnology to enhance bioavailability. Notably, the exploration of oxylipins as therapeutic indicators offers a novel perspective on the molecular mechanisms of TCMs’ cardioprotection. These findings lay a solid foundation for translating traditional herbal wisdom into evidence-based practice, while highlighting the need for future large-scale clinical trials and mechanistic studies. We extend our gratitude to all authors, reviewers, and the editorial team of *Frontiers in Pharmacology* for their invaluable contributions to this Research Topic.
